# Auditory training for tinnitus treatment: a scoping review

**DOI:** 10.1016/j.bjorl.2023.101361

**Published:** 2023-11-17

**Authors:** Anna Carolina Marques Perrella de Barros, Rhayane Vitória Lopes, Daniela Gil, Andreia Cristina Feitosa do Carmo, Ektor Tsuneo Onishi, Fátima Cristina Alves Branco-Barreiro

**Affiliations:** a Universidade Federal de São Paulo (UNIFESP), Escola Paulista de Medicina, Departamento de Fonoaudiologia, São Paulo, SP, Brazil; b Universidade Federal de São Paulo (UNIFESP), Escola Paulista de Medicina, Biblioteca do Campus São Paulo, São Paulo, SP, Brazil; c Universidade Federal de São Paulo (UNIFESP), Escola Paulista de Medicina, Clínica de Zumbido – Departamento de Otorrinolaringologia e Cirurgia de Cabeça e Pescoço, São Paulo, SP, Brazil

**Keywords:** Tinnitus, Audiology, Rehabilitation, Auditory perception

## Abstract

**Objectives:**

Our study aimed to verify the evidence of auditory training employed in the audiological treatment of tinnitus in adults and older adults.

**Methods:**

Scoping review based on a search for articles in journals available in MEDLINE (PubMed), Embase (Elsevier), LILACS (BVS), and Cochrane Library. Titles and abstracts of the retrieved articles were assessed by peers, following the eligibility criteria; they were afterward read in full text, and the references were hand searched in the results found. Studies’ level of evidence was classified into very high (Level A+), high (Level A), moderate (Level B), limited (Level C), low (Level D), or very low (Level D−) based on the Critically Appraised Topics.

**Results:**

2160 records were identified in the searching stage and 15 studies were eligible for data extraction. Study design, sample characterization, auditory training tasks, sound stimuli, outcome measures, and results were extracted. Frequency discrimination training was the most frequent strategy, followed by auditory attentional skills training and multisensory training. Almost all studies with daily auditory training sessions reported significant benefits demonstrated in at least one outcome measure. Studies that used auditory discrimination training and attentional auditory skill stimulation to treat tinnitus obtained quality evidence levels ranging from limited to high (C‒A) and studies that applied multisensory training or attentional training combined with counseling and passive listening in tinnitus patients reached a high-quality evidence level (A).

**Conclusion:**

Recent studies had higher levels of evidence and considered attentional factors and multisensory pathways in auditory training strategies.

## Highlights

• Auditory training for tinnitus is studied as an auditory rehabilitation strategy.
• There is still no consensus on the best practice methodology.
• There is a need for further high-level studies in this area.
• Auditory discrimination training was the most studied type.
• Considering attentional factors and multisensory paths may lead to future research.

## Introduction

Auditory perception of sound in the absence of corresponding external stimuli is described as tinnitus. Factors that affect auditory health can lead to this symptom. Underlying mechanisms are still being studied and include abnormal synchronous neural activity throughout auditory pathway, increasing rates of spontaneous neural discharges in the auditory cortex and subcortical structures, and modifications in the auditory cortex’s tonotopic representation maps.^1^, ^2^, ^3^

It is estimated that 10%–15% of the general population presents tinnitus.^4^, ^5^ In 20% of these, the symptom negatively impacts quality of life, impairing aspects related to sleep, communication, concentration, and social interaction. It is associated with anxiety, irritability, stress, and depression.^5^ A populational study conducted in the city of São Paulo found a 22% prevalence of tinnitus, occurring mostly in females and with advancing age.^6^

Any abnormality that can damage the auditory pathways, various non-auditory conditions, and organic statuses can generate tinnitus.^7^ Hearing loss is related to tinnitus in about 90% of the cases, and hyperacusis may occur in 25%–40%.^4^ Tinnitus is associated with dizziness in cases of Ménière’s disease^8^ and other conditions that simultaneously affect the auditory and vestibular systems.^9^

There is a continuous effort to find alternatives in tinnitus auditory rehabilitation, addressing its heterogeneity of causes and manifestations.^3^, ^10^ Treatment is still challenging in some cases, there is no single and exclusive path for everyone, regarding not only drug therapies or medical procedures but also approaches that involve complementary disciplines.

A systematic review of the existing guidelines to assess and treat tinnitus in adults revealed a consensus in the recommendation of audiological assessments and validated self-assessment questionnaires to investigate stress or suffering generated by the symptom. Documents recommended educational interventions for tinnitus and hearing aids for those with hearing loss.^11^

Sound therapy uses sound stimulation to promote reorganization of the cortex attempting to relieve tinnitus. It has numerous approaches, such as hearing aids and sound generators combined when tinnitus is accompanied by hearing loss, masking to reduce the audibility of tinnitus totally or partially,^10^ and various potential mechanisms of effect, as habituation^12^ and gain reduction.^3^ Customized sound therapy uses a tinnitus management strategy based on the individual’s tinnitus symptoms. Active discrimination training tasks are another form of pitch-based therapy.^3^, ^13^

Auditory training strategies have been used as possible tools in the treatment of perceptual relief from tinnitus.^10^ A previous systematic review investigated the efficacy of auditory perceptual training for tinnitus treatment and revealed the necessity of higher quality evidence on the topic.^13^ It has been thirteen years since this publication, therefore new evidence on auditory training for tinnitus treatment should be investigated.

The question that motivated the present study was: “What is the evidence of auditory training employed in the audiological treatment of tinnitus in adults and older adults?”.

## Methods

This scoping review followed the quality parameters of the Preferred Reporting Items for Systematic Reviews and Meta-Analyses extension for Scoping Reviews (PRISMA-ScR).^14^ Its protocol was published in the Open Science Framework (OSF) under DOI 10.17605/OSF.IO/P9GFY.

### Eligibility criteria

Studies were selected based on the criteria described in Table 1.

**Table 1:** Eligibility criteria.

Inclusion criteria	Types of participants: adults and older adults with tinnitus (older than 18-years); types of intervention: auditory training in patients with tinnitus, with the patient’s active participation, either alone or in combination with other interventions and stimulation modalities; types of study: experimental, quasi-experimental or observational studies in English, Portuguese, and Spanish, encompassing: cross-over studies, ecological studies, longitudinal studies, randomized and non-randomized clinical trials, before-and-after studies, case-control studies, cohort studies, and case series. Besides these, systematic reviews were also considered. No publication date limit was set to retrieve articles.
Exclusion criteria	Studies addressing tinnitus without the therapeutic approach studied, papers exclusively on other therapeutic interventions, and studies that were not available in the database and/or in full text.

### Search

Terms from the Medical Subject Heading (MeSH) vocabulary and free terms were used (Table 2), search was conducted in MEDLINE (PubMed), Embase (Elsevier), LILACS (BVS), and Cochrane Library, and updated until February 2023. Search strategies developed by the authors followed PRESS – Press Review Electronic Search Strategies recommendations.^15^ Sample was selected by convenience, including all studies that met the inclusion criteria.

**Table 2:** Search strategy in the databases.

Databases	Search strategy
PubMed	(“tinnitus”[MeSH Major Topic] AND (“hearing tests”[MeSH Terms] OR (“hearing”[All Fields] AND “tests”[All Fields]) OR “hearing tests”[All Fields] OR (“hearing”[All Fields] AND “test”[All Fields]) OR “hearing test”[All Fields] OR “sound therapy”[Text Word] OR (“psychoacoustical”[All Fields] OR “psychoacoustically”[All Fields] OR “psychoacoustics”[MeSH Terms] OR “psychoacoustics”[All Fields] OR “psychoacoustic”[All Fields]) OR (“otoacoustic emissions, spontaneous”[MeSH Terms] OR (“otoacoustic”[All Fields] AND “emissions”[All Fields] AND “spontaneous”[All Fields]) OR “spontaneous otoacoustic emissions”[All Fields] OR (“otoacoustic”[All Fields] AND “emission”[All Fields]) OR “otoacoustic emission”[All Fields]) OR “auditory training”[Text Word] OR “auditory rehabilitation”[Text Word] OR (“acoustic stimulation”[MeSH Terms] OR (“acoustic”[All Fields] AND “stimulation”[All Fields]) OR “acoustic stimulation”[All Fields]) OR “stimulation training”[Text Word]) AND (“adult”[MeSH Terms] OR “adult”[All Fields] OR “adults”[All Fields] OR “adult s”[All Fields] OR (“aged”[MeSH Terms] OR “aged”[All Fields] OR “elderly”[All Fields] OR “elderlies”[All Fields] OR “elderly s”[All Fields] OR “elderlys”[All Fields]) OR “oldest”[All Fields])) AND (english[Filter] OR portuguese[Filter] OR spanish[Filter]
Embase	‘tinnitus’/exp/mj AND (‘audiometry’/exp OR audiometry OR ‘pure tone audiometry’/exp OR ‘pure tone audiometry’ OR ‘evoked response audiometry’/exp OR ‘evoked response audiometry’ OR ‘speech audiometry’/exp OR ‘speech audiometry’ OR ‘sound therapy’/exp OR ‘sound therapy’) AND (‘adult’/exp OR adult OR ‘aged’/exp OR aged) AND [embase]/lim AND ([english]/lim OR [portuguese]/lim OR [spanish]/lim)
LILACS	(audiometr* OR “sound therapy” OR otoacustic* OR psicoacustic* OR psychoacustic*) AND (db:(“LILACS”)) AND (mj:(“Zumbido”)).
Cochrane Library	Tinnitus AND (“audiologic rehabilitation” OR “sound therapy” OR “audiologic training” OR “hearing test” OR “otoacoustic emission” OR audiometry).

### Selection of sources of evidence

**Figure 1:**
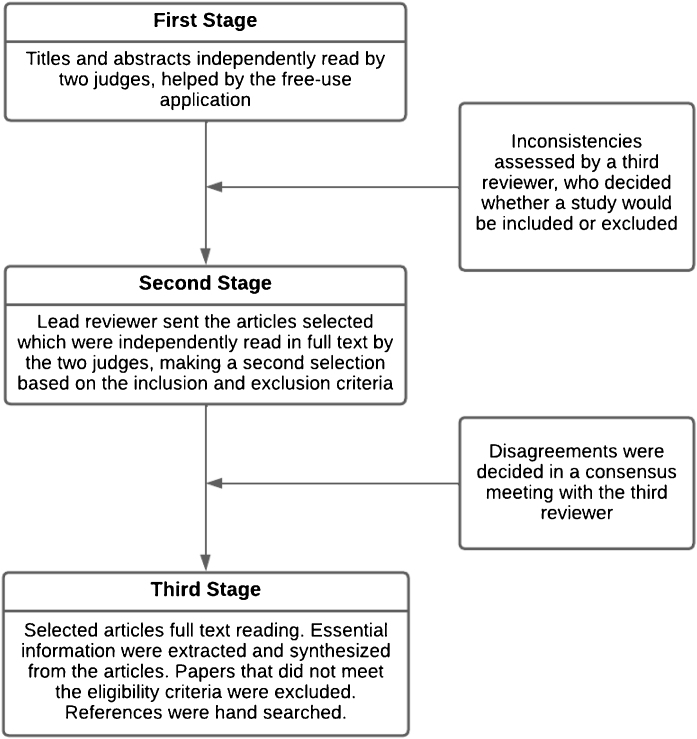
Three-stage process for selection of sources of evidence.

Rayyan QCRI was used to select studies and identify duplicates, in a three-stage process, as described in Fig. 1.

### Data extraction

Data were extracted with an instrument developed for the study and categorized according to the CASP recommendation.^16^ Results were described and summarized according to the objectives of this review and qualitatively assessed based on the Critically Appraised Topics (CAT), which makes critical analyses of the level of evidence of selected articles.^17^

The instrument informs the study design when it is not explicitly reported by the authors and establishes the studies’ level of reliability, considering methodological adequacy (Table 3) and methodological quality (strengths and weaknesses of the study). Studies’ level of reliability was classified into very high (Level A+), high (Level A), moderate (Level B), limited (Level C), low (Level D), or very low (Level D−).

**Table 3:** Classification of methodological adequacy used in the Critically Appraised Topics.

Study design	Level
Systematic review or meta-analysis of randomized controlled studies	AA
Systematic review or meta-analysis of non-randomized controlled and/or before-after studies	A
Randomized controlled study	
Systematic review or meta-analysis of controlled studies without a pretest or uncontrolled study with a pretest	B
Non-randomized controlled before-after study	
Interrupted time series	
Systematic review or meta-analysis of cross-sectional studies	C
Controlled study without a pretest or uncontrolled study with a pretest	
Cross-sectional study (survey)	D
Case studies, case reports, traditional literature reviews, theoretical papers	D−

## Results

**Figure 2:**
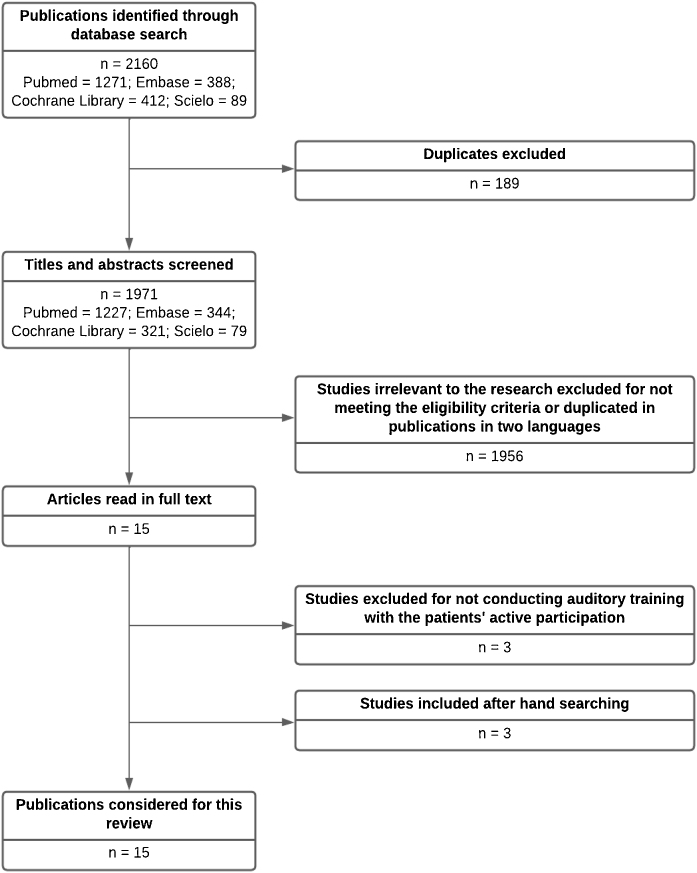
Flowchart of the results.

A total of 2160 articles were identified in databases, 189 duplicates were excluded, leaving 1971 articles, whose titles and abstracts were read. Then, 1956 articles were excluded for not meeting the eligibility criteria, leaving 15 selected papers. These articles were read in full, and three of them were excluded for not meeting the eligibility criteria, leaving 12 articles to be included in this review. The references were hand-searched, and three additional articles were found that met the eligibility criteria, totaling 15 articles. Fig. 2 shows the selection process.

Studies included were published between 2004 and 2022, from New Zealand, Spain, the United Kingdom, Germany, Brazil, Canada, and Australia. Studies were described in detail and categorized as follows: author/date, study design, level (according to qualitative analysis with CAT), sample characterization, auditory training tasks, sound stimuli, outcome measures, and study results (Table 4). Studies and auditory training’s main features were described in Table 5.

**Table 4:** Description of the studies included in the review.

Author/date	Study design	Level	Sample characterization	Auditory training/sound stimuli	Outcome measures	Results	Reliability
(Tugumia et al., 2016)^22^	Randomized controlled clinical trial	A	12 adults, chronic tinnitus (+2 a), normal auditory thresholds or sensorineural hearing loss <55 dB HL (0.25–20 kHz audiometry)	Temporal processing and attentional skill tasks (temporal ordering, temporal resolution, auditory closure, figure-ground, binaural integration, and separation). Eight 40-min weekly sessions, formal auditory training in the sound booth (Study Group) or visual training (Control Group).	THI, GIN (Gaps-in-Noise), frequency pattern test, Speech-in-Noise test, P300 (cognitive potential)	No statistically significant differences were found between the groups before and after auditory and visual training in the analyses of the central auditory processing tests, electrophysiological tests, and THI.	High (90%)
(Wise et al., 2016)^23^	Randomized controlled clinical trial	A	31 adults/older adults, chronic tinnitus (+6 m), moderate non-conductive hearing loss (<80 dB HL, at 0.25–8 kHz)	Sound stimuli matched with tinnitus. 30 min/d, 20 consecutive days, in a computer. Study Group: selective auditory attention training game (identifying target sounds in a virtual auditory setting in detriment of distracting sounds). Control Group: visual game.	TFI, THI, attention tests, auditory evoked potentials	Statistically significant improvement in the TFI and THI indices in the game involving selective auditory attention. Improvement in TFI was correlated with improvement in attention tests and decreased latency in the N1 wave in the auditory evoked potentials.	High (90%)
(Spiegel et al., 2015)^21^	Randomized controlled clinical trial	A	18 adults/older adults, with unilateral tinnitus (onset from 1 to 31 m) and sensorineural hearing loss <80 dB HL.	Multisensory training with visual task combined with tactile and auditory stimulation (tonal stimulus). On a computer, 20-to-30-min daily sessions. Integration Group: three stimuli on the side of tinnitus, with visual cue coherent with this side, and no visual cue when the visual saccade task was on the side opposite to tinnitus. Attention Deviation Group: inversed map, with visual cue and stimulation on the side opposite to tinnitus.	TFI, THI, TSNS, attentional skill assessment (CAB and eye-tracking), DASS.	An overall decrease in TFI scores. Significant decreases in TSNS, with a positive correlation between TFI changes and TSNS scores. No changes were associated with THI or DASS. Statistically significant improvement in attentional skills and eye saccade latency. No difference in the results between the groups.	High (90%)
(Wise et al., 2015)^30^	Non-controlled before-and-after study	C	8 adults/older adults, with chronic tinnitus (+6 m) and bilateral non-conductive hearing loss (auditory thresholds ≥20 dB HL at 0.25–8 kHz and <70 dB HL).	Perceptual training, computerized interactive game, localization, and sound selective attention task. 30 min/d, 20 consecutive days, tinnitus pitch and loudness measured daily before the game. Stimuli at varying frequencies, based on the tinnitus perception frequency. Varying intensity between target and distracting stimuli. Direction of the stimuli randomly established on a horizontal plane (0–315° azimuth).	MML and THI	Significant decrease in post-training total THI score. No statistically significant difference in MML measures.	Limited (70%)
(Hoare et al., 2014)^20^	Randomized controlled clinical trial	A	60 adults/older adults, chronic tinnitus (+6 m), auditory threshold ≥20 dB HL at least one frequency assessed (0.125–14 kHz) in at least one ear.	Frequency discrimination training, standard frequency in the subjects’ normal hearing range, one octave below the hearing loss limit obtained from the audiometric profile, at fixed intensity. Three computer game platforms. Three study groups, varying the game training order. 30 min/d, 5 times a week, 4 weeks in the first game, then 1 week in each of the other games (6-week training).	Qualitative motivation interview assessment, THQ and THI, attention tests	Greater intrinsic motivation in training with the interactive game platform. Changes in tinnitus severity measure indices were not significant. There was no difference between groups in the attention and tinnitus severity measures.	High (90%)
(Hoare et al., 2012)^19^	Randomized controlled clinical trial	A	70 adults/older adults, chronic tinnitus (+6 m), hearing loss >40 dB HL at least one frequency assessed (0.125–14 kHz), in at least one ear.	Auditory frequency discrimination training, computer game platform. Two experiments: 1. Auditory training with stimuli in normal hearing range (standard pure-tone at 2 kHz), standard pure-tone training at 5 kHz (in the frequency range affected by hearing loss); and training with high-band harmonic complex sounds (in the hearing loss frequency range). 30 min/d, 5 times a week, for 4 weeks (20 sessions). 2. Groups trained with stimuli in the normal hearing range (standard pure tone at 2 kHz). 15 min/d, 5 times a week, for 4 weeks (20 sessions); or 60 min, 5 times a week, for 2 weeks (10 sessions).	THQ, psychoacoustic measures (pitch and loudness)	Decreased tinnitus handicap reported in all groups (regardless of stimulus frequency or duration of training), maintained after 1-month follow-up, with no statistically significant difference in the outcome measures. The benefit of auditory training was more generalized, rather than specific, due to determined stimuli or training programs.	High (90%)
(Herraiz et al., 2010)^18^	Randomized controlled clinical trial	A	41 adults, with tonal tinnitus, mild-to-moderate handicap (THI < 56), with onset time ranging from 1 month to 11 years, with hearing loss at high frequencies.	20 min/d, auditory discrimination task, MP3 player, for 30 days. 300 pairs lasting 100 ms in each 10-min track; 70% of pairs with standard tone (4, 6, or 8 kHz), 30% of pairs with frequency-deviation tones based on the standard tone. 100-ms latency between tones in each pair, and 2 s between pairs. 6 tracks, distributed throughout the days of training. Two groups: SAME, (training with stimuli at tinnitus pitch frequency), and NONSAME (training one octave below the tinnitus pitch frequency).	VAS, THI, subjective response	Tinnitus improved in the responses by 42.2%. VAS and THI scores decreased, with a statistically significant difference only in THI. The group trained one octave below the tinnitus pitch frequency significantly decreased in THI, in comparison with the group that trained with stimuli at tinnitus pitch frequency	High (90%)
(Herraiz et al., 2007)^27^	Non-randomized controlled study	B	46 adults, tinnitus (onset from 1 month to 13 years), hearing loss at high frequencies (≥25 dB HL, 4‒8 kHz).	Frequency discrimination training, MP3 player. 20 min/day, 30 days. 400 stimuli lasting 50 ms with random tones, about every 1.5′. 85% broadband noise, 15% pure tone. Five different tracks to be used depending on the day of training (specific protocol for each day of training).	VAS, THI, subjective response	Subjects submitted to training significantly improved the VAS and THI indices and subjective responses, in comparison with controls. Some of the effects observed may be due to the placebo effect or unspecific factors, such as not focusing attention on tinnitus.	Moderate (80%)
(Searchfield et al., 2007)^29^	Non-controlled before-and-after study	C	10 adults/older adults, annoying tinnitus, varying degree of hearing loss from 2 kHz.	Auditory attention and localization tasks, MP3 player. Various auditory stimuli, including common sounds (running water, coughing, barking dog, traffic noise, fax modem, and chattering crowd). 30 min/day, 15 days. Participants should recognize, identify, and write down the auditory object they heard. The sounds were grouped as follows: one in the right ear, one in the left ear, and the other in both, simultaneously.	Psychoacoustic measures (pitch and loudness), MML, and attention tests.	Subjects exposed to auditory training decreased loudness and MML, with a statistically significant difference only in MML. There was a strong correlation between change in performance in one of the attentional tasks (DRT) and change in MML.	Limited (70%)
(Flor et al., 2004)^28^	Non-controlled before-and-after study —	C	12 adults (study group), chronic tinnitus (3 m–6 y), hearing loss (mean of 43.63 dB in the LE and 36.09 in the RE).	Frequency discrimination training, in a computer. Proximal Group: trained with stimuli at a frequency near that of tinnitus. Distal Group: trained with stimuli at a frequency far from that of tinnitus. 2 h/d, 4 weeks. 50% of presentations with different tones; 50% with similar tones.	Tinnitus Questionnaire, MTI, Depression Scale, and Symptom Checklist-90-Revised (SCL-90-R).	Significant decrease in tinnitus severity (group with more training sessions). Significant differences between the first and last day of training (group with more training sessions). Decreased total MTI score and other psychological variables, without statistically significant differences.	Limited (70%)
(Herraiz et al., 2006)^26^	Non-randomized controlled study	B	35 adults/older adults, tinnitus (4 m–12 y), hearing loss >25 dB HL at 4‒8 kHz	Frequency discrimination training, MP3 player. 20 min/d, 30 days. Each training track had 400 randomly mixed tones lasting 50 ms, about every 1.5 s. 85% were white-noise tones, 15% 4 kHz pure tones. Five different tracks, in varying presentations according to the day.	VAS, THI, and subjective question about tinnitus improvement	43% reported improved tinnitus when asked. The improvement reported in the question and VAS was statistically significant in the training group, in comparison with the waiting-list group. Improved THI indices, though not statistically significant.	Moderate (80%)
(Searchfield et al., 2021)^24^	Randomized controlled study	A	20 adults/older adults, predominantly unilateral chronic tinnitus (+6 m), hearing loss at high frequencies (lower than 70 dB HL).	Multisensory perceptual training, in a computer. Visual task combined with auditory and tactile stimulation. 30 min/d, 20 days. Study Group: use of a low dose of fluoxetine. Control Group: use of placebo. Tonal stimuli (120 ms), matched with the subjective tinnitus pitch measure. Tactile stimuli with a brief vibration on the temple (120 ms). Participants were instructed to gaze at a point ahead on the center of the computer screen, make a saccade to the right or left when the format of the point changed, and hit a key on the computer corresponding to the side of the saccade. Auditory stimulus in a congruent standard (tactile, visual, and auditory stimuli on the dominant side of tinnitus).	TFI, THI, 5 tinnitus classification scales, with scores ranging from 0 to 10 (areas: loudness, discomfort, disturbance, ability to ignore it, and annoyance), resting-state functional magnetic resonance imaging (Rs-fMRI), and DASS.	A significant change in loudness, disturbance, and tinnitus-related problem scores. No significant changes were found in the THI, TFI, and DASS measures. Fluoxetine use did not change the behavioral outcome measures. Significant changes in neural connectivity patterns were identified after training with Rs-fMRI. Positive correlation between changes in sensory and attentional neural networks and significant changes in tinnitus classification.	High (90%)
(Searchfield and Sanders, 2022)^25^	Randomized controlled study	A	61 adults/older adults, constant moderate-severe tinnitus (+6 m), hearing loss with a maximum of a moderate degree.	Attentional training through a Tinnitus Calibration Task and an Auditory Object Identification and Localization (AOIL) task delivered by an app designed for the study. Participants were encouraged to move their attention away from tinnitus and had different everyday sounds presented monaurally or binaurally. The Study Group also used Bluetooth bone-conduction headphones, a neck pillow speaker, and a cloud-based clinician dashboard to communicate with researchers and personalization the app during the process. Control Group used a known app with passive sound therapy (White Noise Lite). 2 h/d, 12 weeks.	TFI, rating scales, the Client Oriented Scale of Improvement in Tinnitus (COSIT), System Usability Scale (SUS) and mHealth App Usability Questionnaire (MAUQ).	TFI’s changes after 6 and 12 weeks of treatment were greater in the Study Group compared to the Control Group. Rating scales showed more significant differences in the Study Group. COSIT scores were better at Study Group. These changes were not statistically significant. Usability measures were similar for both groups.	High (90%)
(Hoare et al., 2010)^13^	Systematic review	A	NR	NR	NR	Great variability in tinnitus severity between studies, the character of the training tasks, and stimuli. Information on methodological issues is insufficient. There is no standardized procedure for outcome measures; questionnaires or psychoacoustic measures were used. Only one randomized clinical trial; no study reported using blinding on the part of the participants or examiners. There was no association between tinnitus sensation measure and severity measure; little control of the beneficial effects of placebo or unspecific factors.	High (90%)
(Herraiz et al., 2009)^31^	Qualitative study	D−	NR	NR	NR	The following were described: phenomena involved in auditory plasticity due to sensory deprivation after sensorineural hearing loss, effects of audiological rehabilitation and auditory training on the auditory system. Tinnitus as a result of cortical reorganization was discussed. Then the description of and reasons for choosing auditory discrimination training to manage tinnitus. The results of some cited studies suggest a positive effect of frequency discrimination training on tinnitus, tending to better responses when the training frequencies are close but not similar to tinnitus pitch. Further clarification is still needed regarding which is the best stimulus frequency and duration of training — i.e., the best protocol to be used.	Very low (55%)

≥, Greater than or equal; >, greater than; <, less than; m, months; years ago; min/day, minutes per day; h, hours; ms, milliseconds; s, seconds; dBHL, decibel hearing level; Hz, hertz; kHz, thousand hertz; RE, right ear; LE, left ear; THI, tinnitus handicap inventory; TFI, tinnitus functional index; THQ, tinnitus handicap questionnaire; MTI, multidimensional tinnitus inventory; TSNS, tinnitus severity numeric scale; CAB, comprehensive attention battery; VAS, Visual Analogue Scale; MML, Minimum Masking Level; DRT, discriminate reaction time test; DASS, depression, anxiety and stress scale; COSIT, client oriented scale of improvement in tinnitus.

**Table 5:** Main features of auditory training for tinnitus patients’ studies and strategies.

Studies’ design	Randomized controlled clinical trials (n = 8)^18^, ^19^, ^20^, ^21^, ^22^, ^23^, ^24^, ^25^; non-randomized controlled studies (n = 2)^26^, ^27^; before-and-after non-controlled studies (n = 3)^28^, ^29^, ^30^; systematic review and meta-analysis (n = 1)^13^; and qualitative study (n = 1)^31^
Studies’ reliability	Ranged from very low (55%)^31^ to high (90%)^13^, ^18^, ^19^, ^20^, ^21^, ^22^, ^23^, ^24^, ^25^
Sample	Chronic tinnitus patients^19^, ^20^, ^22^, ^23^, ^24^, ^25^, ^28^, ^30^; criteria ranging from 3 (three)^28^ (n = 1) to 6 (six) months^19^, ^20^, ^23^, ^24^, ^25^, ^30^ (n = 6); participants with tinnitus for at least 4 (four) months^26^ or at least 2 (two) years.^22^ Number of participants ranged from 8 to 70.
Participants	Adults (aged 18 years or older) with hearing loss^18^, ^19^, ^20^, ^21^, ^22^, ^23^, ^24^, ^25^, ^26^, ^27^, ^28^, ^29^, ^30^; older adults (subjects aged 60 years or older)^19^, ^20^, ^21^, ^23^, ^24^, ^25^, ^26^, ^29^, ^30^
Training’s type	Frequency discrimination training^18^, ^19^, ^20^, ^26^, ^27^, ^28^, ^31^; stimulation of temporal and attentional processing skills (n = 1)^22^; selective auditory attention alone (n = 1)^23^ or in combination with sound localization tasks (n = 3)^25^, ^29^, ^30^; multisensory stimulation (n = 2)^21^, ^24^
Sound stimuli	Pure tone,^18^, ^19^, ^20^, ^21^, ^22^, ^24^, ^26^, ^27^, ^28^ alone^18^, ^20^, ^28^ or in combination with noise^19^, ^26^, ^27^; distorted or competing verbal stimuli^22^; everyday sounds^29^; customized sounds matched with the psychoacoustic perception of tinnitus^23^, ^30^; a target tinnitus avatar sound and everyday sounds.^25^
Auditory training’s period	15-days^29^; 20-days^21^, ^23^, ^24^, ^30^; 1-month^18^, ^19^, ^26^, ^27^, ^28^; 1 and a half month^20^; 2-months^22^; 3-months.^25^
Training sessions’ period	30-min^19^, ^20^, ^21^, ^23^, ^24^, ^28^, ^30^; 10-min^26^, ^27^; 20-min^18^; 40-min^22^; 2-hs of cumulative use per day of the app.^25^
Training sessions’ frequency	Daily^18^, ^21^, ^23^, ^24^, ^26^, ^27^, ^28^, ^29^; five sessions a week^19^, ^20^; once a week.^22^
Auditory training tools	Computerized platforms (n = 7)^19^, ^20^, ^21^, ^23^, ^24^, ^28^, ^30^; MP3 players;^18^, ^26^, ^29^ acoustic booth^22^; smartphone app designed.^25^
Outcome measures	Questionnaires combined with other measures^18^, ^19^, ^21^, ^22^, ^23^, ^24^, ^25^, ^26^, ^27^, ^28^; psychoacoustic measures^19^, ^29^, ^30^ such as tinnitus pitch and loudness (n = 2)^19^, ^29^ and Minimum Masking Level (MML) (n = 2);^29^, ^30^ attentional skills tests (n = 4)^20^, ^21^, ^29^, ^30^; participants’ subjective impression (n = 6)^18^, ^19^, ^24^, ^25^, ^26^, ^27^; electrophysiological measures of hearing (P300)^22^, ^23^; central auditory processing behavioral assessment^30^; resting-state functional magnetic resonance imaging^21^; Visual Analog Scale (VAS)^26^, ^27^; tinnitus classification scales^24^; numeric rating scales.^25^

A review produced by Hoare et al.^13^ encompassed studies with active hearing tasks of various kinds and diverging stimuli. The study by Searchfield et al. associated multisensory perceptual training with drug therapy to verify whether the drug would enhance the effects of training.^24^ Searchfield and Sanders^25^ used attentional auditory training with stimulus to move attention away from tinnitus in auditory space and to attend to different locations or sounds responding to prompts.

Eight studies^18^, ^21^, ^23^, ^24^^,^^26^, ^28^, ^29^, ^30^ reported statistically significant improvements in at least one of the outcome measures. Four studies^19^, ^20^, ^22^, ^25^ did not find statistically significant effects of the auditory training. Tinnitus Handicap Questionnaire (THI) was the tool with the most changes in post-intervention administration,^18^, ^28^, ^31^ followed by attentional skill tests,^21^, ^23^ Tinnitus Functional Index (TFI),^21^, ^30^ VAS,^26^, ^27^ and Client Oriented Scale of Improvement in Tinnitus (COSIT).^25^

Improvement after auditory training was reported in tinnitus classification scales^24^ and queries developed by the authors,^18^, ^26^, ^27^ as well as greater intrinsic motivation to train in interactive game platforms.^20^ One study mentioned a decrease in psychoacoustic measures of loudness and Minimum Masking Level (MML) after auditory training.^29^

Hoare et al.^19^ observed generalized benefits after auditory training, which could not be ascribed to a specific stimulus or auditory training program. Searchfield et al.,^24^ using functional imaging, verified changes in the connectivity pattern of the sensory and attentional neural networks after multisensory perceptual training, with no additional effect due to medication.

Frequency discrimination training was the type of auditory training with the most changes in the outcome measures,^18^, ^26^, ^27^, ^28^ followed by selective auditory attentional training,^23^, ^25^, ^29^, ^30^ localization,^25^, ^29^, ^30^ and multisensory attentional training, which involved auditory, tactile, and visual stimuli.^21^

Herraiz et al.^31^ presented the essentials of frequency discrimination training as a treatment for tinnitus. Frequency discrimination training could partially reverse changes in tonotopic representation due to peripheral damage and improve tinnitus, making it a new therapeutic option to be developed.

A systematic review^13^ verified low to moderate evidence quality levels on auditory training interventions studies. Few randomized controlled clinical trials provided impartial and generalizable evidence, which would prove that the perceptual auditory training had a relevant clinical effect on tinnitus.

Our research shows new studies focused on the topic^19^, ^20^, ^21^, ^22^, ^23^, ^24^, ^25^, ^30^ after this review and quality of evidence improvement since the majority were randomized controlled trials,^9^, ^20^, ^21^, ^22^, ^23^, ^24^, ^25^ high-quality evidence level (A). Studies published after 2010 included procedures that ranged from frequency discrimination training (n = 2),^19^, ^20^ auditory training tasks to stimulate the temporal and attentional processing skills (n = 1),^22^ stimulation of selective auditory attention alone (n = 1)^23^ or associated with sound localization tasks (n = 2),^25^, ^30^ to the multisensory stimulation through auditory, tactile, and visual stimulus (n = 2).^21^, ^24^

## Discussion

Auditory-perceptual training can be defined as the auditory nervous system learning to hear with the active involvement of sounds, in which listeners learn to make certain distinctions in what they systematically hear.^32^ The auditory system is reorganized in response to changes in auditory input. This system is responsible for a complex mechanism composed of distributed nervous networks that integrate to represent auditory stimuli, due to auditory system plasticity.^33^

The term “sound therapy” has been generally and indiscriminately used to refer to the use of any sound in tinnitus treatment. However, the interaction mechanisms between input sound and tinnitus are heterogeneous, which is why maskers, hearing aids, and other stimulations are used with different sound changes, which are described in the literature to treat tinnitus.^10^

Our study defined the patients’ active participation as one of the inclusion criteria to investigate the evidence of auditory training as a therapeutic strategy for tinnitus. There are other audiological approaches to treat tinnitus that involve passive sound stimulation, more suitable to sound therapy strategies, rather than auditory training. Auditory training involves active listening to various auditory stimuli to improve auditory skills and trigger neuroplasticity.^34^

Auditory rehabilitation may cause changes in the auditory system, leading to brain reorganization mechanisms, affecting auditory performance as well.^35^ Perceptual learning effects are accompanied by neural plasticity in the brainstem and cortex. Inferior colliculus plays an important role in auditory learning. Auditory system plasticity effectively occurs in young adults. In older adults, this brain mechanism is also present, with change possibilities associated with improved communication in challenging hearing situations.^36^

Duration and frequency of auditory training varied in the selected studies. Except for one study, which conducted one 40-min session a week (totaling 8 weeks) of auditory training in a sound booth,^22^ all other studies had at least five sessions a week. Daily training sessions were the most used frequency. The minimum stimulation time per day was 20 min, for 15–30 days. Almost all studies with daily auditory training sessions reported significant benefits demonstrated in at least one outcome measure.^18^, ^21^, ^23^, ^24^^,^^26^, ^27^, ^28^, ^29^ Searchfield and Sanders^25^ did not find statistical differences, but results showed clinical effects of intervention.

Learning mechanisms must be established for auditory training to have relevant effects on increased neural plasticity and consequent changes in this system. Auditory training increases the area of cortical representation, changing these areas of the central nervous system regarding certain stimuli.^35^

The period of stimulation may have an impact on plastic changes, influenced by the time and frequency of training to which the subject is exposed. In our study, daily training proved to be more effective in the auditory training of subjects with tinnitus, regardless of the time of stimulation, which ranged from 15 to 30 days.

Based on the hypothesis that selective serotonin-reuptake inhibitors could potentialize cortical plasticity in adults, use of fluoxetine was combined with the training strategy; however, there was no additional effect related to the drug administration along with training.^24^

Tinnitus is strongly associated with symptoms of anxiety and depression. Severity of tinnitus and the subjects’ predisposition to develop anxiety and depression are important factors that influence this association.^37^ Only three papers^21^, ^24^, ^28^ investigated anxiety and depression symptoms in their outcome measures and there was no statistically significant difference in these measures before and after training.

Psychoacoustic measures can determine the perception characteristics in tinnitus frequency (pitch) and intensity (loudness) and verify the possibility of masking and its post-masking suppression, as in the assessment of residual inhibition effect, confirming that these sounds interact with the mechanism that generates tinnitus.^37^

Three studies^19^, ^23^, ^29^ used psychoacoustic measures to assess tinnitus as outcome measures, establishing tinnitus pitch and loudness^19^, ^29^ and MML.^29^, ^30^ MML was the only psychoacoustic measure that showed a statistical difference after the auditory training intervention.^29^ Modulating tinnitus with acoustic stimulation, as in tests that assess MML and tinnitus residual inhibition, may furnish prognostic data on the long-term effects of the treatments involving auditory stimulation.^38^

There is methodological variability in the clinical and scientific psychoacoustic measures assessment protocols.^39^ The lack of standardized protocols hinders comparisons and may lead to diversified findings in the measures.

Auditory Discrimination Training (ADT) was described in the literature based on the capacity to change brain tonotopic representation due to central nervous system plasticity. There is no consensus on the frequencies used as stimuli to obtain the best results in terms of relief from tinnitus perception. Studies considered hearing loss’ frequency range, tinnitus pitch, or the tinnitus spectrum to define the stimuli used inside or outside these ranges.

In the present study, frequency discrimination was the type of auditory training that showed the most changes in outcome measures.^18^, ^26^, ^27^, ^28^ Lack of standardization in tinnitus pitch mapping techniques proved to be a critical factor in treatments based on this measure, which can make some therapies unfeasible.^20^ Lack of consensus on the most effective strategy for sound stimuli, whose frequencies are or are not close to the one that corresponds to the tinnitus pitch, may complicate the procedures in ADT.^10^ It was supposed that some widespread benefits from this approach should be related to other mechanisms such as selective attention or emotional state.^19^

Assessing auditory training methods for patients with tinnitus that used attentional auditory skill stimulation,^22^, ^23^, ^25^, ^29^^,^^30^ only two of them^22^, ^25^ did not have statistically significant results in their outcome measures. However, the first one used a standard auditory training program for central auditory processing disorders that stimulated auditory skills in general^22^ and the other had clinically significant changes in the intervention that include the training.^25^ Other three studies with specific strategies aimed at tinnitus found improvements in at least one outcome measure.^23^, ^29^, ^30^

Subjects with tinnitus and normal hearing performed worse in tasks involving auditory attention skills and tinnitus might be associated with low performance in selective and sustained auditory attention.^40^ Attentional auditory skills stimulation may be an interesting path to be explored in future research addressing audiological therapeutic management of tinnitus, adapting, and customizing it to issues involved in processes underlying tinnitus considering current scientific understanding.

Considering that the consequences of tinnitus to the central nervous system would involve plastic reorganization encompassing auditory and non-auditory areas of the brain and that multisensory perceptual learning is usually more consistent than unimodal learning, multisensory training combined multimodal stimuli as a tinnitus treatment method.^21^ Also, combined therapeutic strategies like auditory attentional training, counseling, and passive auditory stimulation were developed aiming to modify tinnitus-related neural networks.^24^

In the present study, two papers applied multisensory stimulation, combining auditory, tactile, and visual stimuli. There was a limited though statistically significant improvement in the subjective measures of tinnitus in the population studied, showing that multisensory attention training may be an effective tool to manage tinnitus.^21^ Resting-state functional magnetic resonance imaging found neuroplastic changes in neural connectivity after multisensory perceptual training, which showed the involvement of auditory and non-auditory cortical and subcortical areas in subjective chronic tinnitus.^24^

Auditory training programs are often available on game platforms and need to be interesting enough to ensure the patients’ adherence. An advantage of computerized programs is that it facilitates training at home while hearing health professionals are regularly visited to monitor the effects of training.^34^ In our review, seven studies^19^, ^20^, ^21^, ^23^, ^24^, ^28^^,^^30^ used computer-based auditory training in patients with tinnitus and one developed an app that provides auditory attentional training combined with other strategies.^25^ One study verified greater intrinsic motivation with the interactive game platform.^20^

The CAT methodological qualitative assessment classified the papers included in our results into varying reliability levels, ranging from very low (55%) to high (90%). Eight randomized controlled studies^18^, ^19^, ^20^, ^21^, ^22^, ^23^, ^24^, ^25^ obtained Level A in study design analysis and high reliability.

The studies that used auditory discrimination training^18^, ^19^, ^20^, ^26^, ^27^, ^28^, ^31^ and attentional auditory skill stimulation^22^, ^23^, ^29^, ^30^ applied to tinnitus patients obtained quality evidence levels ranging from limited to high (C‒A) whereas the studies that applied multisensory training^21^, ^24^ or attentional auditory training combined with passive listening and counseling^25^ in tinnitus subjects got a high-quality evidence level (A).

A systematic review conducted on the topic in 2010 found a scarcity of randomized controlled clinical trials, whose levels of evidence ranged from low to moderate.^13^ There has been a positive qualitative change over the years in publications involving auditory training in patients with tinnitus, as there are currently more studies with higher methodological levels.

Our results revealed frequency discrimination training was the most studied auditory discrimination training type applied to tinnitus patients. However, most of the studies on this topic are dated before 2010. Further research incorporated other methodologies at the study design. The scientific work seems to get the other hand and the latest publications have considered attentional factors and multisensory paths, that can be correlated to tinnitus physiopathology, at the training program activities.

Most papers in the literature (which seek to customize the protocols used in tinnitus treatment with sounds) considered a specific dimension – e.g., using tinnitus pitch as a reference measure – to individually adapt tinnitus treatment, disregarding the complex combination of different dimensions in tinnitus. Therapy customization and planning tools should be included and assessed when treating such patients.^10^

Although the quality of the studies improved in the last decade, a great challenge in auditory training for tinnitus treatment is to carry out methodologically replicable research. There is currently no predefined way for the auditory treatment of subjects with tinnitus aiming to improve the perception of the symptom and relieve the discomfort related to it. Another challenge is the number of research participants. Treatment takes time and the participants’ active participation; hence, longitudinal follow-up with a significant sample and a consistent effect size has not yet been accomplished.

Along with these factors, methodological heterogeneity in the outcome measures is an issue that reflects current audiological assessment in the field. There are various non-homogeneous protocols, including in the methodology of the subjective tests used as tinnitus audiological assessment instruments.

Studies are not yet comprehensive, with limited samples and variable methodologies. This hinders the comparison of auditory training effects and generalization of the results to the general population with tinnitus, which would validate their clinical applicability.

## Conclusion

Auditory discrimination training was the most studied approach. Recent studies had higher levels of evidence and considered attentional factors and multisensory pathways in auditory training strategies. Further large-scale research is necessary, using adapted strategies directed to the topic, considering current scientific knowledge.

## Funding

The authors declare no funding.

## Conflicts of interest

The authors declare no conflicts of interest.
